# Real-world study of adverse events associated with ceftazidime/avibactam based on the U.S. Food and Drug Administration adverse event reporting system database

**DOI:** 10.3389/fcimb.2026.1698293

**Published:** 2026-01-22

**Authors:** Xin Zheng, Yang Peng, Yang-xue Ou, Bi-bo You, Lu Zhang, Yuan Cheng, Mo Cheng, Pu Xiang

**Affiliations:** 1 Department of Pharmacy, Dianjiang People’s Hospital of Chongqing, Chongqing, China; 2 Department of Pharmacy, Chongqing Dianjiang County Hospital of Traditional Chinese Medicine, Dianjiang, Chongqing, China

**Keywords:** adverse events, ceftazidime/avibactam, combination, FAERS, respiratory failure, safety signals

## Abstract

**Objective:**

Ceftazidime/avibactam (CZA), a combination of the third-generation cephalosporin ceftazidime and the novel, non-β-lactam β-lactamase inhibitor avibactam, was widely used in the treatment of multidrug-resistant (MDR) Gram-negative bacterial pathogens. In light of the growing prevalence of MDR Gram-negative bacterial infection, it is imperative to gain a deeper understanding of the true extent of adverse events (AEs) associated with CZA.

**Methods:**

AE reports primarily associated with CZA were retrieved from the Food and Drug Administration Adverse Event Reporting System (FAERS) database from the first quarter of 2015 to the first quarter of 2025, and all AEs were extracted. AE signal detection was conducted using the reporting odds ratio (ROR), proportional reporting ratio (PRR), Bayesian confidence propagation neural network (BCPNN), and multi-item gamma Poisson shrinker (MGPS) methods. Additionally, a multivariable logistic regression analysis was performed to evaluate the safety of CZA combined with meropenem, explicitly adjusting for confounders such as age, sex, and concomitant nephrotoxins.

**Results:**

A total of 2,444 AE reports with CZA as the preferred suspected drug were obtained, identifying 89 preferred terms (PTs) involving 24 system organ classes (SOCs). The ratio of males to females was approximately two times higher in all cases, with the highest number of reports originating from China. Some significant AE signals have been revealed by four methods, including renal and urinary disorders (*n* = 112, ROR 2.49, PRR 2.43, EBGM 2.43, IC 1.28) and hepatobiliary disorders (*n* = 98, ROR 5.04, PRR 4.88, EBGM 4.88, IC 2.29). Regarding combination therapy with meropenem, multivariable analysis revealed a specific safety signal: the risk of respiratory failure remained significantly elevated [adjusted OR (aOR) 3.26, *P* = 0.038] independent of baseline severity. Conversely, acute kidney injury showed no significant association (aOR 1.07, *P* = 0.868), suggesting that the respiratory risk is pharmacodynamically driven rather than a result of generalized clinical deterioration.

**Conclusion:**

The present study identified significant and novel AEs signals for CZA. Notably, the specific association between the CZA-meropenem combination and respiratory failure warrants vigilant clinical monitoring, distinct from general disease progression risks.

## 1 Introduction

Multidrug-resistant (MDR) Gram-negative bacterial infections, especially those caused by carbapenemase-producing pathogens, including carbapenem-resistant *Acinetobacter baumannii*, *Pseudomonas aeruginosa*, and *Enterobacteriaceae*, not only significantly prolong the length of hospitalization and increase the economic burden of patients but also result in a significantly higher mortality rate, which has become a major challenge for global public health (Torres et al., 2018; Mills and Marchaim, 2021; Almangour et al., 2022). Ceftazidime/avibactam (CZA) is a novel β-lactam/β-lactamase inhibitor combination that effectively enhances the antimicrobial activity of the third-generation, extended-spectrum cephalosporin ceftazidime against multidrug-resistant bacteria (Yahav et al., 2020). The observed effect can be attributed to the inhibitory effect of avibactam on a broad spectrum of activity, which inhibits Ambler class A, class C, and certain class D β-lactamases (Shields et al., 2017; Shirley, 2018). CZA has been approved for marketing in the US, EU, and China since 2015. Subsequent to its emergence as a pivotal pharmaceutical agent, it has been utilized as a primary treatment for complicated intra-abdominal infections, complicated urinary tract infections, hospital-acquired pneumonia, including ventilator-associated pneumonia, and other severe infections caused by MDR Gram-negative bacteria (Carmeli et al., 2016; van Duin and Bonomo, 2016; Wagenlehner et al., 2016). It is currently recommended as a preferred treatment option for carbapenem-resistant Enterobacteriaceae (CRE) and refractory *Pseudomonas aeruginosa* infections by clinical guidelines in multiple nations.

CZA has been on the market for 10 years, and there has been a paucity of studies conducted on its safety. A more in-depth evaluation of their long-term and comprehensive safety characteristics in the real world is particularly urgent. The U.S. Food and Drug Administration Adverse Event Reporting System (FAERS) is one of the world’s largest, most comprehensive, and publicly available databases for post-marketing drug safety monitoring (Sakaeda et al., 2013). The system functions as a significant data resource for pharmacovigilance research by collecting reports of adverse drug events voluntarily submitted by healthcare professionals, patients, pharmaceutical companies, and regulatory agencies (Morris et al., 2024). FAERS has become an important tool for the detection of rare or unanticipated adverse drug events due to its broad coverage, large volume of data, and ability to detect potential safety signals at an early stage. In recent years, several studies based on the FAERS database have gradually revealed possible safety issues with a variety of drugs in the real world (Gatti et al., 2021; Liu et al., 2024). In 2023, Qi et al. employed the FAERS database to investigate the adverse reactions of CZA, thereby identifying several potential AE signals, including cholestasis, drug-induced liver injury, hepatocellular injury, and hemolytic anemia (Zhang et al., 2024). In addition to the adverse reactions already mentioned in the drug inserts, the use of CZA may be associated with a series of new and potentially serious adverse events (Yao et al., 2024). However, the investigation did not delve further into the subgroup results, nor did it take into account the impact of drug combinations on AEs for the prevalence of combination regimens centered on CZA in the context of CRE treatment. Therefore, it is imperative to gain a deeper understanding of the true extent of AEs associated with CZA.

The present study aims to systematically analyze all adverse events reported for CZA since its introduction to the market. The FAERS database will be utilized as a pharmacovigilance tool, and various well-established proportional imbalance analysis methods will be applied. Through the controlled analysis of age and gender subgroups, differences in AE risks among different populations and differences in AEs associated with the most common drug combinations can be assessed, thereby providing a scientific basis for individualized medication.

## 2 Materials and methods

### 2.1 Data source and collection

**Figure 1:**
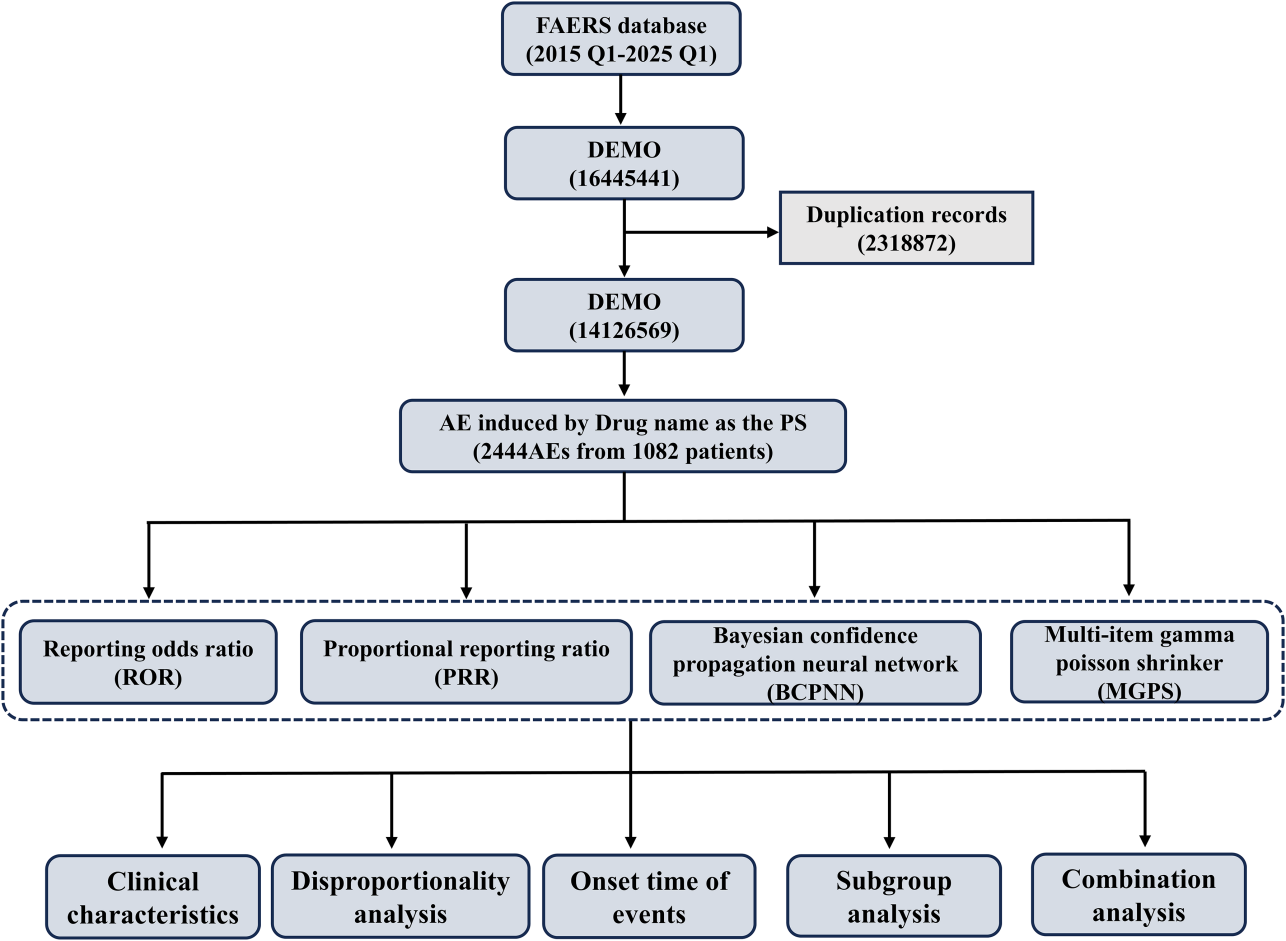
Flow chart of data extraction and analysis. A detailed description of the flow chart for data extraction and analysis of adverse events for CZA in the United States Food and Drug Administration Adverse Event Reporting System (FAERS).

The data extraction and analysis procedure are shown in **Figure 1**. The data were sourced from the FAERS database. This study utilized the raw adverse event report data in ASCII format obtained from the website, covering a total of 41 quarters from the first quarter of 2015 to the first quarter of 2025. The MySQL database management system was used to store, manage, and integrate the database, which contains information such as drug-related adverse events, adverse event reporting dates and outcomes, routes of administration, dosages, cumulative dosages, patient gender and age, and reporting countries. Reported drugs in FAERS are categorized into four groups: Primary Suspect (PS), Secondary Suspect (SS), Concomitant (S), and Interacting (I). Both the brand name and the generic name are employed to identify records related to ceftazidime/avibactam. The search terms include “Avibactam,” “Avycaz,” “Emblaveo,” and “Zavicefta.” In this study, we focused exclusively on data that designated CZA as the ‘prime suspect’. Subsequently, we categorized and characterized AEs in accordance with the preferred terminology (PT) and the system organ classification (SOC) within the International Medical Dictionary for Regulatory Activities (MedDRA 28.0 Edition). This research was carried out and documented in strict adherence to the recommendations outlined in the READUS-PV guidelines. The complete READUS-PV checklist can be accessed in **Supplementary Table S1**. The data sources are publicly accessible, thus eliminating the need for ethical approval.

### 2.2 Data processing

The data was sourced from multiple quarterly database tables, covering various dimensions such as patient information, medication details, adverse event reports, indications, and outcome data (Shu et al., 2022). The integration of information from disparate database tables into a unified table was achieved by employing SQL queries. The queries employed *INNER JOIN* and *LEFT JOIN* operations to relate data across different tables while retaining all necessary fields. The *INNER JOIN* was used to join the demographic information table with the drug information table. The target drugs were filtered from the drug table. The *LEFT JOIN* integrated the AE information, indication information, and outcome information tables with the main data table to retrieve complete patient records. Invalid or flagged records were removed from the dataset. The records under scrutiny were obtained from deletion tables that were published on a quarterly basis by the FDA. The *UNION* operations were utilized to consolidate deletion tables across multiple quarters into a unified list of records designated for removal. The consolidated list was utilized to eliminate invalid records from the merged primary dataset, thereby generating a refined dataset. The cleaned data table was then validated by checking record counts to ensure the correctness of the cleaning process. The number of records in the cleaned dataset was compared with the expected count. In this study, Python 3.9 was used to execute SQL scripts for data processing tasks.

### 2.3 Statistical analysis

Disproportionality analysis was utilized to characterize adverse event reports associated with CZA by comparing them against all other reported drugs within the FAERS database. This analysis typically comprises two components, including frequentist statistics and Bayesian statistics. Frequentist statistics includes the reporting odds ratio (ROR) and proportional reporting ratio (PRR), while Bayesian statistics encompasses the multi-item gamma Poisson shrinker (MGPS) and Bayesian confidence propagation neural network (BCPNN) (Evans et al., 2001; van Puijenbroek et al., 2002; Norén et al., 2013). The algorithm is based on the fourfold table shown in **Table 1**, and the signal mining method and its signal generation conditions are shown in **Table 2**. In this study, AEs that met the positive thresholds for all four methods (ROR: lower limit of 95% CI > 1, PRR: χ2 S4, lower limit of 95% CI > 1, EBGM: EBGM05 > 2, BCPNN: IC025 > 0) were regarded as potential AE signals of CZA. The integration of the ROR, PRR, MGPS, and BCPNN algorithms is a strategy employed to leverage the strengths inherent in multiple methodologies, thereby mitigating the potential bias that may arise from reliance on a solitary algorithm. To strictly control for potential confounding by indication, a multivariable logistic regression analysis was performed. The ROR was adjusted (aROR) by including covariates such as age, sex, reporting year, and concomitant use of nephrotoxic agents (vancomycin and aminoglycosides) as proxies for disease severity. A p-value < 0.05 was considered statistically significant.

**Table 1:** Four grid table.

Category	CZA-related AEs	Non-CZA-related AEs	Total
CZA	a	b	a + b
Other drugs	c	d	c + d
Total	a + c	d + b	a + b + c + d

AEs, adverse events; a, number of reports containing both the target drug and target adverse drug reaction; b, number of reports containing other adverse drug reaction of the target drug; c, number of reports containing the target adverse drug reaction of other drugs; d, number of reports containing other drugs and other adverse drug reactions.

**Table 2:** ROR, PRR, BCPNN, and MGPS methods, formulas, and thresholds.

Algorithms	Equation	Criteria
ROR	ROR = ad/b/c	lower limit of 95% CI > 1, N ≥ 3
	95% CI = e^ln(ROR) ± 1.96(1/a+1/b+1/c+1/d)^0.5^	
PRR	PRR = a (c + d)/c/(a+b)	PRR ≥ 2, χ2 ≥ 4, N ≥ 3
	χ^2^ = [(ad-bc)^2](a+b + c + d)/[(a + b) (c + d) (a + c) (b + d)]	
BCPNN	IC = log_2_a (a+b + c + d) (a + c) (a + b)	IC025 > 0
	95%CI = E (IC) ± 2V(IC)^0.5	
MGPS	EBGM = a (a+b + c + d)/(a + c)/(a + b)	EBGM05 > 2
	95% CI = e^ln(EBGM) ± 1.96(1/a+1/b+1/c+1/d)^0.5^	

a, number of reports containing both the target drug and target adverse drug reaction; b, number of reports containing other adverse drug reaction of the target drug; c, number of reports containing the target adverse drug reaction of other drugs; d, number of reports containing other drugs and other adverse drug reactions. 95% CI, 95% confidence interval; N, the number of reports; χ^2^, chi-squared; IC, information component; IC025, the lower limit of 95% CI of the IC; E (IC), the IC expectations; V (IC), the variance of IC; EBGM, empirical Bayesian geometric mean; EBGM05, the lower limit of 95% CI of EBGM.

## 3 Results

### 3.1 Descriptive characteristics on the AE reports

A total of 16,445,441 AE reports were registered in the FAERS database from 1 April 2015 to 30 June 2025. These reports involved 1,082 patients and 2,444 AEs, as shown in **Table 3**. Among all AEs, the proportion of AEs in men (47.4%) was higher than that in women (26.9%). The age distribution showed that 18.9% (204) of elderly patients were over 60 years old, while 15.2% (164) were aged 18–60 years. However, 63.4% (686) of patients had missing ages. The vast majority of the AEs were reported in patients from China (25.9%), followed by the United States (12.0%) and France (9.2%). Most reports (67.9%) were provided by physicians and health professionals.

**Table 3:** Clinical characteristic of reports with CZA from the FAERS database (2015 to 2025).

Characteristics	Case numbers	Characteristics	Case numbers
Number of events	1082	Reporter	
Gender		Physician	538 (49.7%)
Male	513 (47.4%)	Health professional	197 (18.2%)
Female	291 (26.9%)	Consumer	166 (15.3%)
Miss	278 (25.7%)	Pharmacist	143 (13.2%)
Age		Other health-professional	31 (2.9%)
<18	28 (2.6%)	Miss	7 (0.6%)
18–60	164 (15.2%)	Reporting year	
60–80	145 (13.4%)	2015	13 (1.2%)
>80	59 (5.5%)	2016	25 (2.3%)
Missing	686 (63.4%)	2017	65 (6.0%)
**Reporting countries (TOP 5)**		2018	75 (6.9%)
China	280 (25.9%)	2019	137 (12.7%)
United States	130 (12.0%)	2020	117 (10.8%)
France	100 (9.2%)	2021	124 (11.5%)
Italy	81 (7.5%)	2022	120 (11.1%)
Spain	57 (5.3%)	2023	162 (15.0%)
		2024	188 (17.4%)
		2025	56 (5.2%)

### 3.2 Signal AE mining and analysis of AE signals at the PT level

A total of 24 kinds of SOCs were classified according to MedDRA for the organs and systems involved. Analysis using the ROR, PRR, BCPNN, and EBGM methods identified 87 PT signals, involving 24 SOCs. The signal strengths of the reports associated with CZA at the SOC level are presented in **Table 4**. The most frequent SOCs were general disorders and administration site conditions (507, 20.7%), injury poisoning, and procedural complications (299, 12.2%), and infections and infestations (290, 11.9%). Important SOCs that met the four criteria were renal and urinary disorders (112, ROR 2.49, PRR 2.43, EBGM 2.43, IC 1.28) and hepatobiliary disorders (98, ROR 5.04, PRR 4.88, EBGM 4.88, IC 2.29). Furthermore, the top 30 AEs were identified, ranked by the number of AE reports, and 23 AEs with significant safety signals were found using four algorithms at the PT level (**Table 5**). In our study, the highest number of CZA AEs was death, followed by pathogen resistance, septic shock, decreased platelet count, renal failure, and acute kidney injury. It is important to note that our data mining revealed several important AEs not explicitly mentioned in the specifications for CZA, including multiple organ dysfunction syndrome, respiratory failure, hypotension, and others. Our analysis identified additional AEs that contribute to an enhanced comprehension of the safety profile of CZA.

**Table 4:** The signal strength of AEs of CZA at the SOC level in FAERS database.

System organ class (SOC)	Case	ROR (95%)	PRR(χ2)	EBGM(EBGM05)	IC(IC025)
General disorders and administration site conditions	507 (20.7%)	1.20(1.09–1.32)	1.16 (13.54)	1.16(1.05)	0.21(0.1)
Injury, poisoning and procedural complications	299 (12.2%)	1.03(0.92–1.17)	1.03 (0.28)	1.03(0.91)	0.04(−0.1)
Infections and infestations	290 (11.9%)	2.35(2.08–2.66)	2.19 (197.94)	2.19(1.94)	1.13(0.9)
Investigations	276 (11.3%)	2.08(1.84–2.36)	1.96 (137.47)	1.96(1.73)	0.97(0.8)
Nervous system disorders	208 (8.5%)	1.10(0.95–1.27)	1.09 (1.72)	1.09(0.95)	0.13(−0.1)
Skin and subcutaneous tissue disorders	124 (5.1%)	0.89(0.74–1.06)	0.89 (1.65)	0.89(0.75)	−0.16(−0.4)
Renal and urinary disorders*	112 (4.6%)	2.49(2.06–3.01)	2.43 (95.64)	2.43(2.01)	1.28(1.0)
Gastrointestinal disorders	102 (4.2%)	0.48(0.40–0.59)	0.50 (54.34)	0.50(0.41)	−0.99(−1.3)
Hepatobiliary disorders*	98 (4.0%)	5.04(4.12–6.17)	4.88 (304.82)	4.88(3.99)	2.29(1.9)
Blood and lymphatic system disorders	79 (3.2%)	2.02(1.61–2.52)	1.98 (39.24)	1.98(1.59)	0.99(0.6)
Respiratory, thoracic and mediastinal disorders	77 (3.2%)	0.68(0.54–0.85)	0.69 (11.50)	0.69(0.55)	−0.54(−0.9)
Psychiatric disorders	65 (2.7%)	0.49(0.38–0.63)	0.50 (33.66)	0.50(0.39)	−0.99(−1.3)
Cardiac disorders	45 (1.8%)	0.86(0.64–1.16)	0.86 (0.97)	0.86(0.64)	−0.21(−0.6)
Metabolism and nutrition disorders	35 (1.4%)	0.70(0.50–0.97)	0.70 (4.54)	0.70(0.50)	−0.51(−1.0)
Vascular disorders	32 (1.3%)	0.68(0.48–0.96)	0.68 (4.89)	0.68(0.48)	−0.56(−1.0)
Neoplasms benign, malignant and unspecified	19 (0.8%)	0.26(0.17–0.41)	0.27 (38.79)	0.27(0.17)	−1.89(−2.5)
Immune system disorders	17 (0.7%)	0.58(0.36–0.94)	0.59 (5.03)	0.59(0.36)	−0.77(−1.4)
Musculoskeletal and connective tissue disorders	17 (0.7%)	0.13(0.08–0.21)	0.14 (97.69)	0.14(0.08)	−2.87(−3.5)
Eye disorders	14 (0.6%)	0.29(0.17–0.48)	0.29 (24.77)	0.29(0.17)	−1.78(−2.5)
Surgical and medical procedures	7 (0.3%)	0.20(0.09–0.41)	0.20 (23.05)	0.20(0.09)	−2.34(−3.2)
Ear and labyrinth disorders	7 (0.3%)	0.66(0.31–1.38)	0.66 (1.25)	0.66(0.31)	−0.60(−1.6)
Product issues	7 (0.3%)	0.15(0.07–0.32)	0.16 (32.28)	0.16(0.07)	−2.67(−3.5)
Congenital, familial and genetic disorders	5 (0.2%)	0.78(0.33–1.88)	0.78 (0.30)	0.78(0.33)	−0.35(−1.5)
Pregnancy, puerperium and perinatal conditions	2 (0.1%)	0.21(0.05–0.86)	0.22 (5.74)	0.22(0.05)	−2.22(−3.4)

Asterisks (*) indicate statistically significant signals in four algorithms; ROR, reporting odds ratio; PRR, proportional reporting ratio; EBGM, empirical Bayesian geometric mean; EBGM05, the lower limit of the 95% CI of EBGM; IC, information component; IC025, the lower limit of the 95% CI of the IC; CI, confidence interval; AEs, adverse events.

**Table 5:** The signal strength of adverse events of CZA at the PTs level in FAERS database.

PT	Case	ROR (95% Cl)	PRR(χ2)	EBGM (EBGM05)	IC(IC025)
Death*	163 (6.7%)	5.10(4.35–5.98)	4.83(501.12)	4.82(4.12)	2.27(2.0)
Pathogen resistance*	105 (4.3%)	61.64(50.67–74.97)	59.03(5973.41)	58.83(48.59)	5.88(5.61)
Septic shock*	32 (1.3%)	19.34(13.64–27.42)	19.10(548.84)	19.09(13.46)	4.25(3.1)
Platelet count decreased*	28 (1.1%)	6.69(4.61–9.71)	6.62(133.85)	6.62(4.56)	2.73(1.9)
Renal failure*	27 (1.1%)	4.98(3.41–7.28)	4.94(84.97)	4.94(3.38)	2.30(1.6)
Pyrexia	27 (1.1%)	1.98(1.35–2.89)	1.97(12.90)	1.97(1.35)	0.98(0.4)
Acute kidney injury*	27 (1.1%)	4.64(3.17–6.78)	4.60(76.22)	4.60(3.15)	2.20(1.5)
Blood creatinine increased*	25 (1.0%)	9.60(6.48–14.24)	9.52(190.65)	9.51(6.41)	3.25(2.3)
Multiple organ dysfunction syndrome*	24 (1.0%)	24.07(16.10–35.98)	23.84(524.84)	23.82(15.93)	4.57(3.1)
Renal impairment*	22 (0.9%)	6.80(4.47–10.35)	6.75(107.92)	6.75(4.44)	2.75(1.8)
Diarrhea	21 (0.9%)	0.84(0.55–1.29)	0.84(0.65)	0.84(0.55)	−0.25(−0.9)
Encephalopathy*	20 (0.8%)	21.03(13.54–32.66)	20.86(378.03)	20.85(13.42)	4.38(2.8)
Respiratory failure*	19 (0.8%)	6.56(4.18–10.30)	6.51(88.77)	6.51(4.15)	2.70(1.7)
Seizure*	19 (0.8%)	4.38(2.79–6.88)	4.36(49.19)	4.35(2.77)	2.12(1.3)
Pneumonia	19 (0.8%)	1.51(0.96–2.37)	1.50(3.21)	1.50(0.96)	0.59(−0.1)
Rash	19 (0.8%)	1.14(0.72–1.79)	1.14(0.32)	1.14(0.72)	0.19(−0.5)
Hepatic function abnormal*	19 (0.8%)	13.38(8.52–21.02)	13.29(215.87)	13.28(8.45)	3.73(2.4)
Sepsis*	17 (0.7%)	3.86(2.40–6.22)	3.84(35.80)	3.84(2.38)	1.94(1.0)
Erythema	16 (0.7%)	1.98(1.21–3.23)	1.97(7.67)	1.97(1.20)	0.98(0.2)
Thrombocytopenia*	16 (0.7%)	3.68(2.25–6.01)	3.66(30.98)	3.66(2.24)	1.87(1.0)
Cholestasis*	15 (0.6%)	20.16(12.13–33.50)	20.04(271.27)	20.03(12.05)	4.32(2.5)
Hepatic enzyme increased*	15 (0.6%)	5.88(3.54–9.77)	5.85(60.39)	5.85(3.52)	2.55(1.4)
Drug-induced liver injury*	15 (0.6%)	13.76(8.28–22.87)	13.68(176.31)	13.68(8.23)	3.77(2.2)
Delirium*	14 (0.6%)	10.47(6.19–17.71)	10.42(119.17)	10.41(6.16)	3.38(1.9)
White blood cell count decreased	13 (0.5%)	3.02(1.75–5.22)	3.01(17.52)	3.01(1.75)	1.59(0.6)
Blood bilirubin increased*	13 (0.5%)	11.98(6.94–20.66)	11.92(130.00)	11.91(6.91)	3.57(2.0)
Pruritus	13 (0.5%)	0.92(0.53–1.59)	0.92(0.09)	0.92(0.53)	−0.12(−0.9)
Neurotoxicity*	12 (0.5%)	18.47(10.47–32.58)	18.38(197.16)	18.37(10.42)	4.20(2.2)
Depressed level of consciousness*	11 (0.5%)	7.07(3.91–12.78)	7.04(57.04)	7.04(3.89)	2.82(1.4)
Hypotension	11 (0.5%)	1.39(0.77–2.51)	1.39(1.19)	1.39(0.77)	0.47(−0.4)
Coma*	11 (0.5%)	5.84(3.23–10.55)	5.81(43.87)	5.81(3.21)	2.54(1.2)

Asterisks (*) indicate statistically significant signals in four algorithms; PT, preferred term; ROR, reporting odds ratio; PRR, proportional reporting ratio; EBGM, empirical Bayesian geometric mean; EBGM05, the lower limit of the 95% CI of EBGM; IC, information component; IC025, the lower limit of the 95% CI of the IC; CI, confidence interval.

### 3.3 Time to onset of CZA-associated AEs

**Figure 2:**
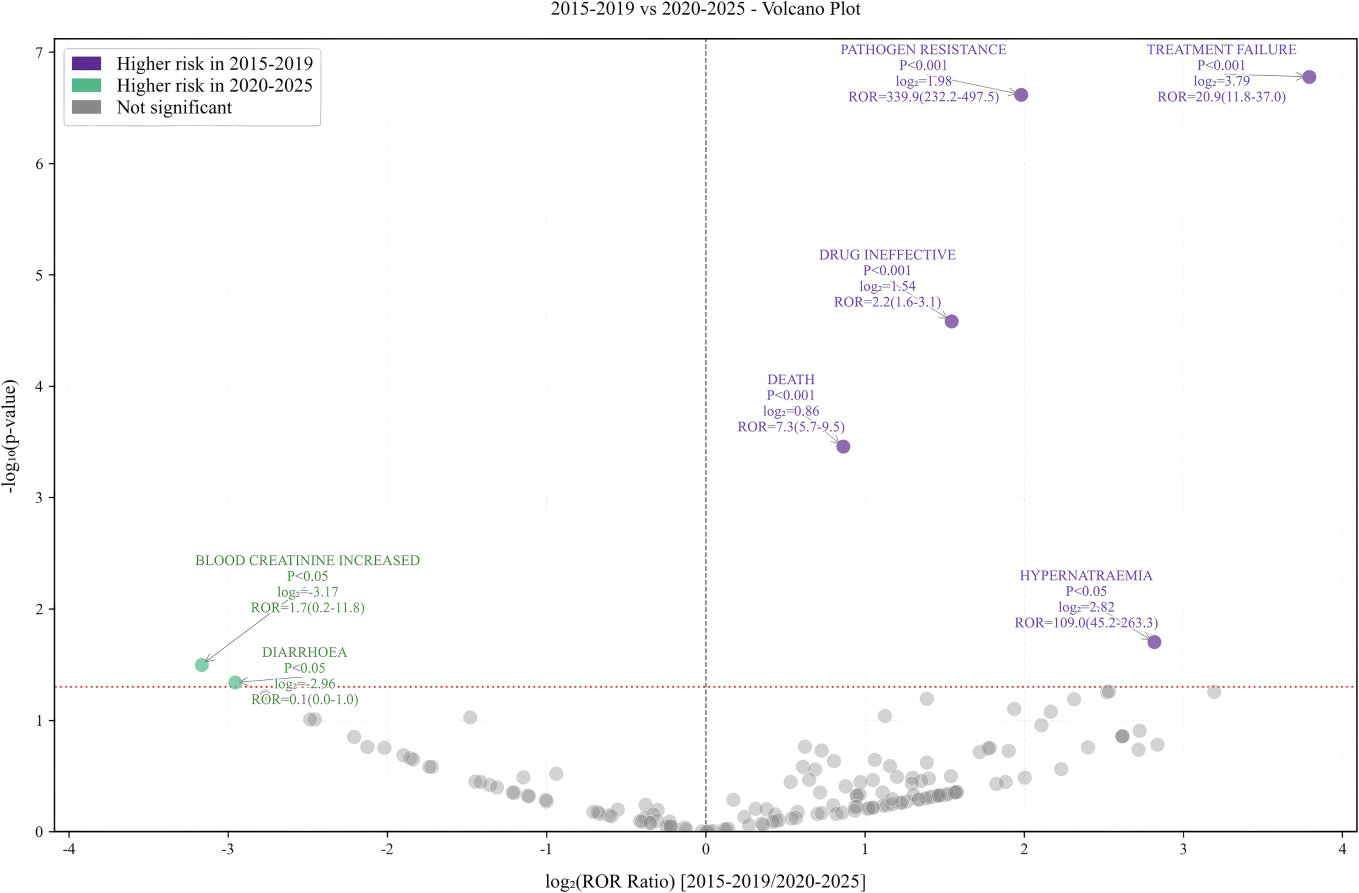
Volcano plot related to AEs from 2015 to 2019 and 2020 to 2025.

**Figure 3:**
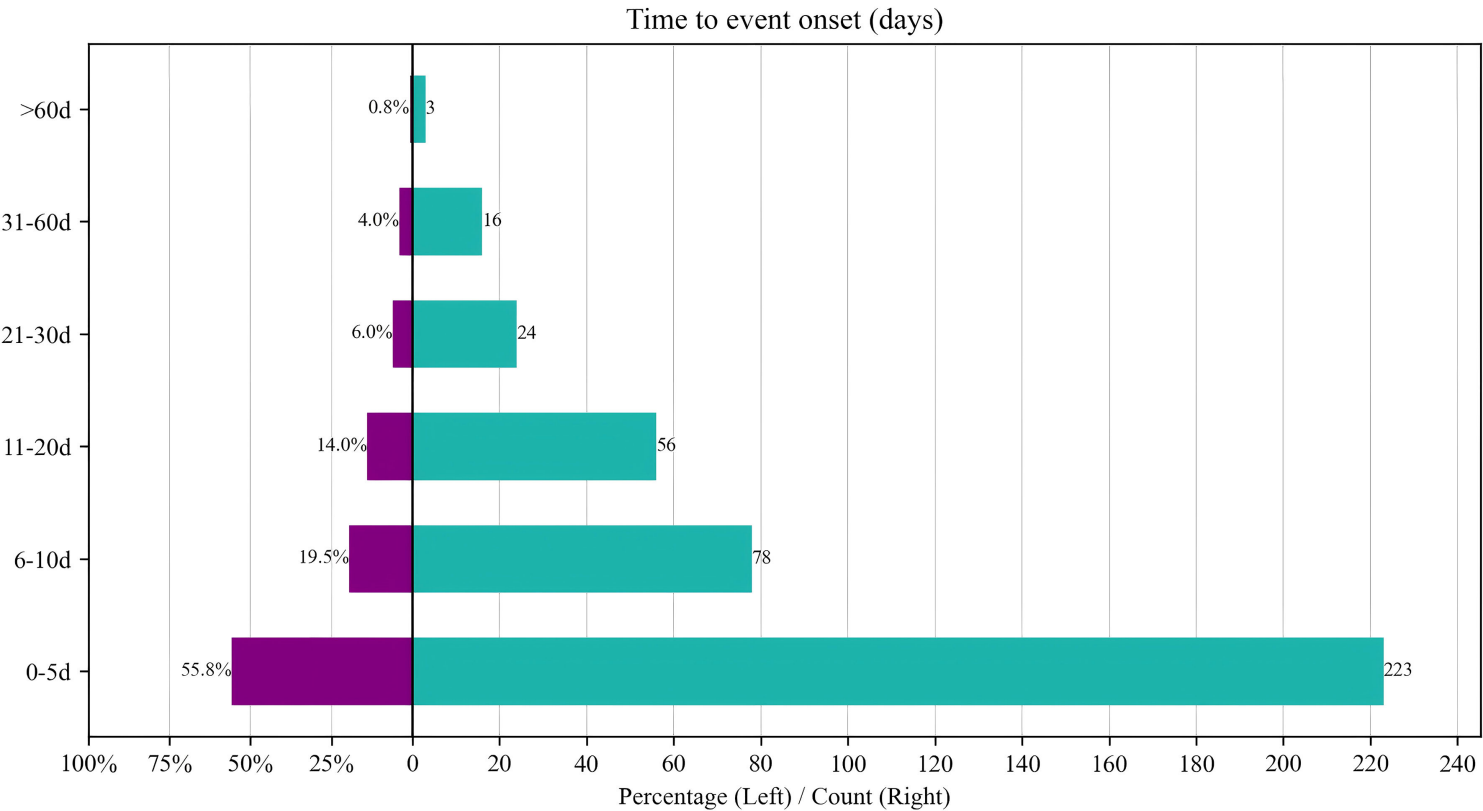
Time to onset of CZA-related AEs.

**Figure 4:**
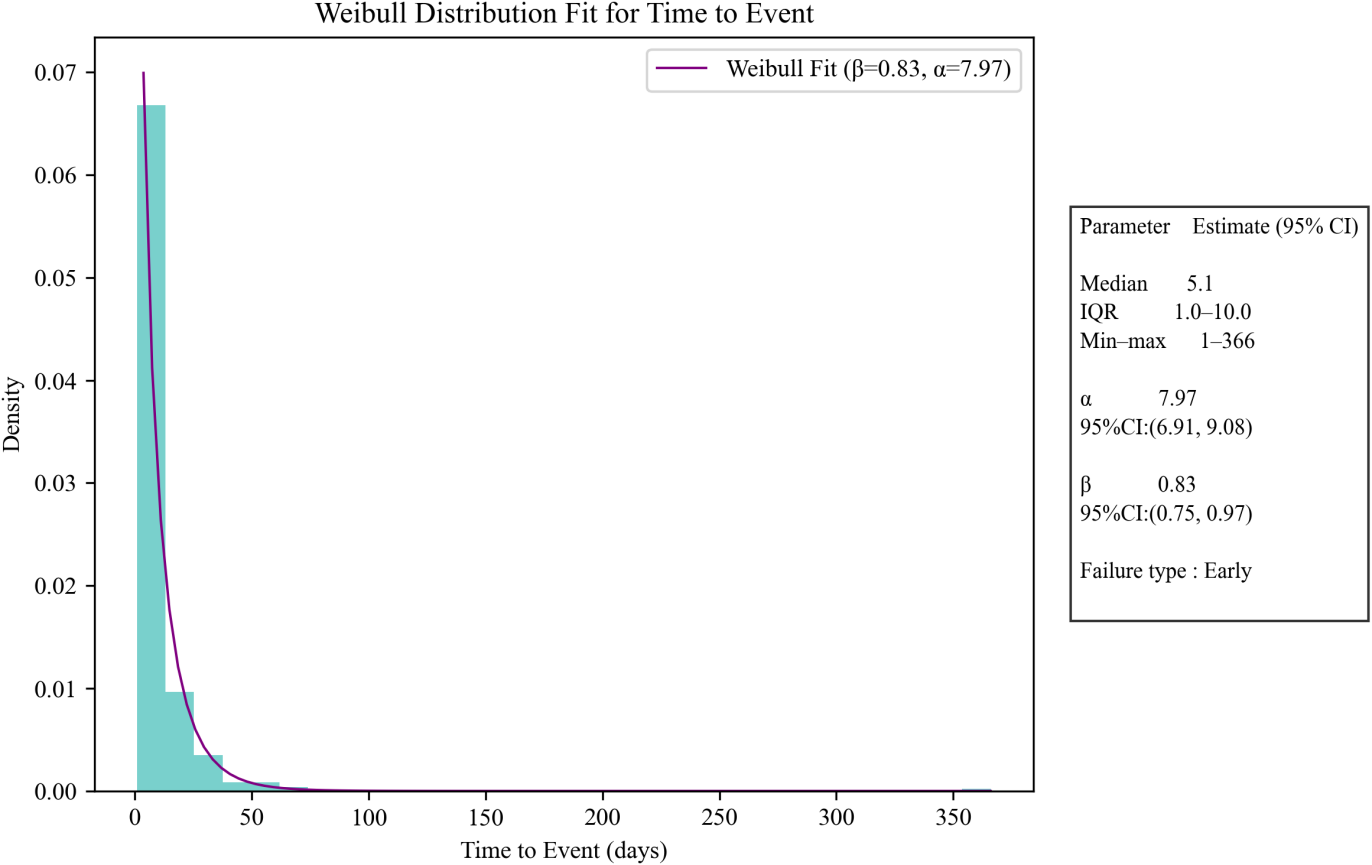
Time to onset analysis of AEs after treatment with CZA.

Based on the time period of AE, we observed that from 2015 to 2020, the risk of CZA-induced nausea and elevated serum creatinine events remained relatively low. However, after 2020, there was a significant increase in the risks of pathogen resistance, treatment failure, drug ineffectiveness, death, and hypernatremia. Of all reported AEs, 400 contained complete and accurate detailed information on the timing of the occurrence. The median time to AE onset was 5.1 days (IQR 1–10 days), and it can be seen that most AEs (223, 55.8%) occurred within the first 5 days of CZA use as shown in **Figures 2** and **3**. In the assessment of Weibull 3-parameter analysis (**Figure 4**), the shape parameter β and its upper limit of 95% CI were found to be less than 1, indicating a decreasing probability of AEs over time. It is noteworthy that a small minority of 4.8% of AEs occurred 30 days after the commencement of CZA treatment. These findings underscore the significance of monitoring patients for potential AEs during the initial 10 days of CZA treatment and throughout the entire treatment process.

### 3.4 Safety of CZA according to age and gender

**Figure 5:**
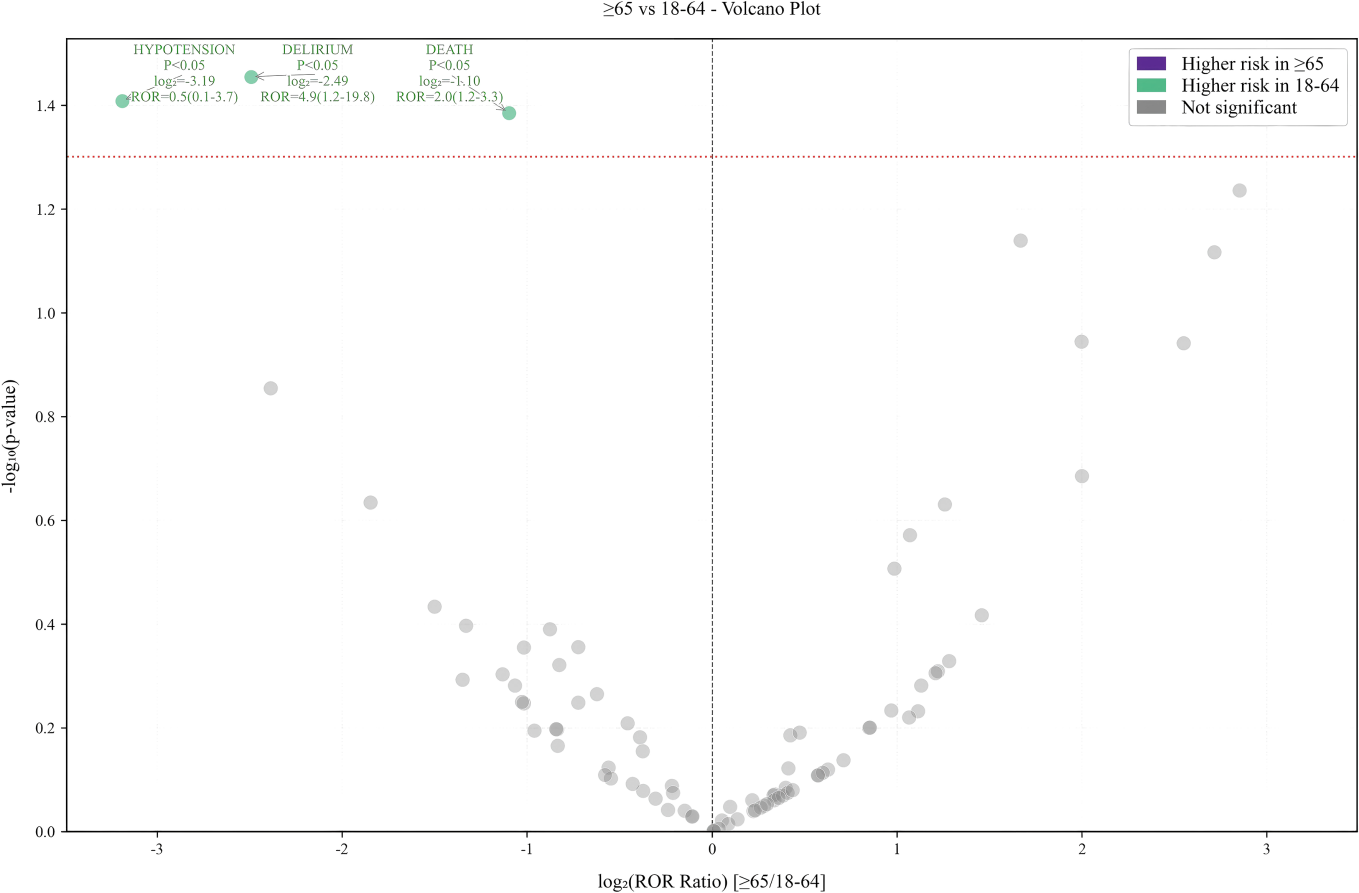
Age subgroup analysis.

**Figure 6:**
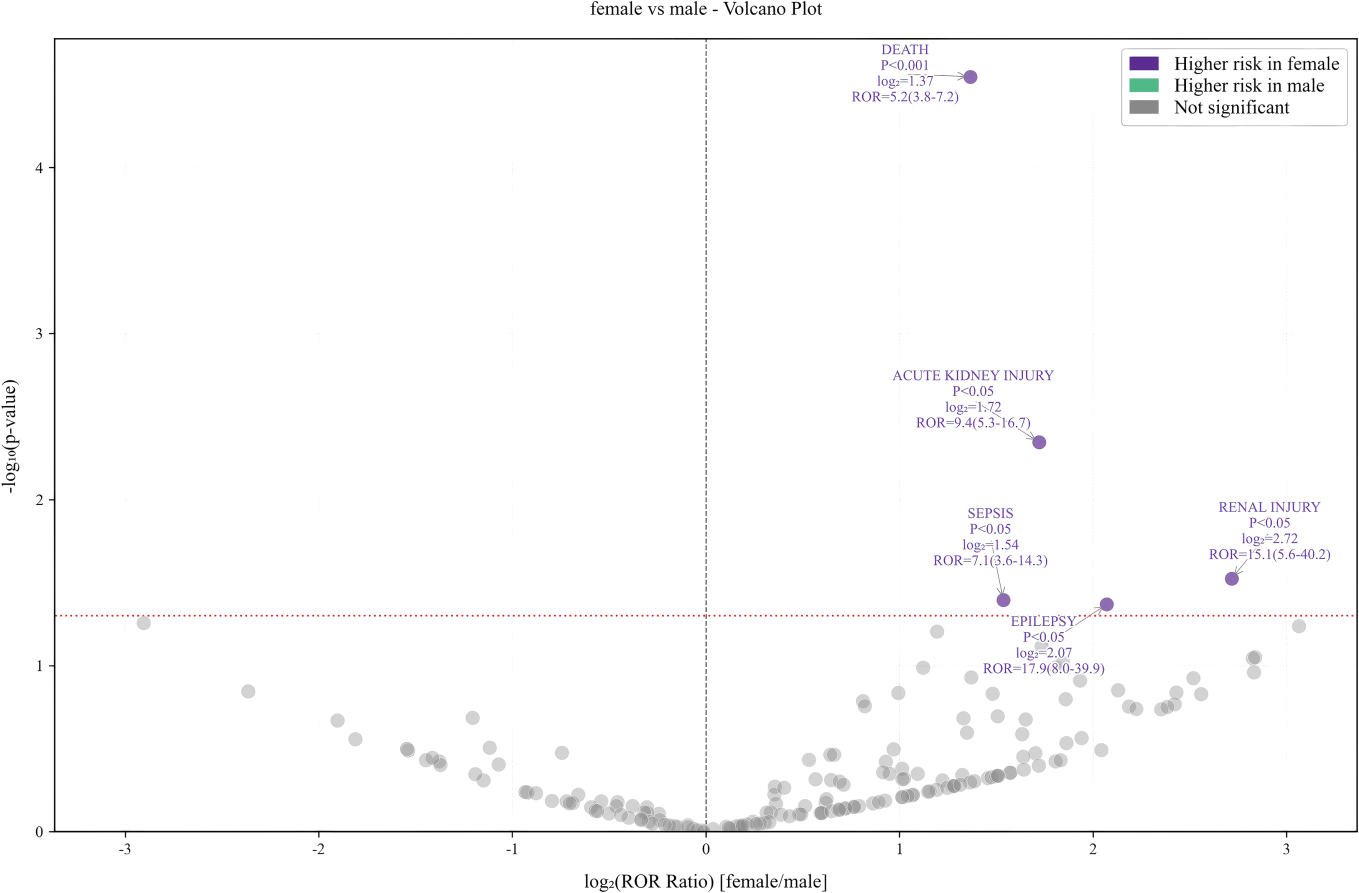
Gender subgroup analysis.

Subgroup analyses were conducted to mitigate the influence of confounding factors related to demographic characteristics. The PT that had the highest number of reported cases was death and hypernatremia in the 18- to 64-year-old and >64-year-old subgroups, respectively. However, there were no significant differences between gender subgroups, with death being the most common AE. The results for the various subgroups are displayed in a volcano plot, where events are negatively correlated on the left side of the x-axis, indicating a lower incidence of PT in that subgroup, and positively correlated on the right side of the x-axis, denoting a higher incidence of PT in that subgroup, as shown in **Figures 5** and **6**. In the age subgroup, the risk of hypotension, delirium, and death was found to be lower after the administration of CZA to subjects aged between 18 and 64 years. In the gender-based subgroup analysis, the female population exhibited an elevated risk of mortality, acute kidney injury, renal failure, sepsis, and epilepsy. This information is critical for clinical management and can guide clinical decision makers in developing personalized treatment plans based on subgroup characteristics.

### 3.5 Safety of CZA versus CZA in combination with meropenem

**Figure 7:**
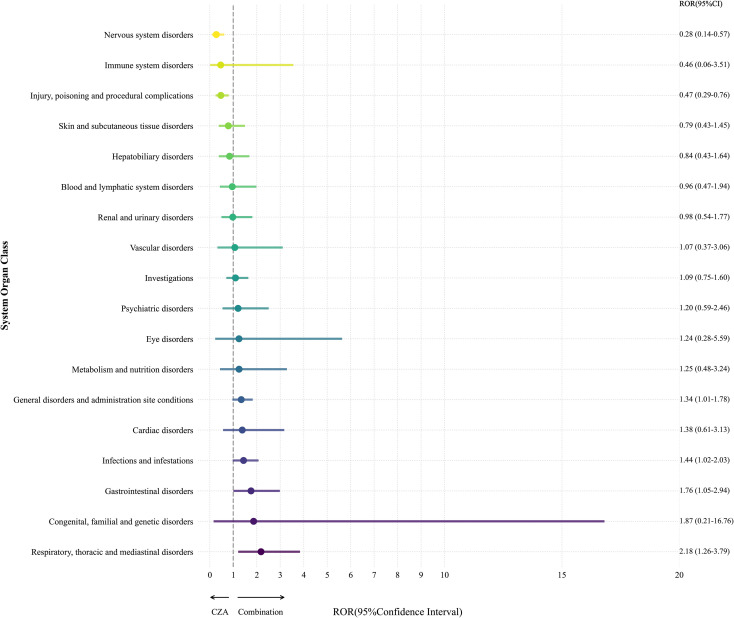
Disproportionately reported SOC for CZA versus CZA and meropenem in the FAERS database. The forest plot shows ROR with 95% confidence intervals.

CZA is recommended for use alone in KPC enzyme-producing CRE and in combination with other drugs in metallo-β-lactamase–producing CRE infections and mixed infections. Therefore, we analyzed the use of combination therapy further to investigate whether the AEs identified were due to drug interactions. Based on the frequency of combination use and the categories suspected, the primary drugs used in combination were meropenem, tigecycline, aztreonam, and vancomycin. We found that when CZA was used alone, the risks of neurological disorders, injury, poisoning, and procedural complications were lower. However, when CZA was combined with meropenem, the risks of general disorders and administration site conditions, infections and infestations, gastrointestinal disorders, and respiratory, thoracic, and mediastinal disorders were higher, as shown in **Figure 7**.

In the multivariable logistic regression analysis (**Supplementary Table S2**), the risk of respiratory failure remained significantly elevated in the combination group (aOR 3.26, 95% CI [1.07–9.93], *P* = 0.038), supporting a pharmacodynamic interaction. Crucially, no significant association was observed for acute kidney injury (AKI) (aOR 1.07, *P* = 0.868). This dissociation suggests that the respiratory signal is a specific drug effect rather than a proxy for generalized clinical deterioration. Conversely, the persistent signals for death and septic shock likely reflect residual confounding by indication, driven by the critical baseline status of patients requiring salvage therapy.

## 4 Discussion

CZA offers important treatment options for MDR Gram-negative bacterial infections. The scarcity of preclinical data makes it crucial to collect pharmacovigilance data from post-marketing systems where AEs are reported, which could greatly improve drug quality standards. This study explored the risk status of its AEs from the perspective of signaling risk by accessing CZA data from the FAERS database and performing signal mining.

We observed that AE reports have shown an overall upward trend from 2015 to 2025, with the number of recently recorded AEs continuing to increase, which may reflect the efficacy of CZA in the treatment of infections caused by drug-resistant bacteria. The study results highlight the importance of continuous monitoring of AEs. We compared the clinical characteristics of patients treated with CZA. The incidence of AEs was significantly higher in male patients (47.4%) than in female patients (26.9%). Middle-aged and young adults were the primary reporting population, accounting for the majority of cases (18–60 years: 15.2%), while the elderly population aged 80 years and above was relatively small, primarily due to the absence of age data in most cases (63.4%).

### 4.1 Basic information

Our consistency analysis indicates that CZA was associated with significant AEs in 24 SOC categories, including general disorders and administration site reactions, injuries, poisoning, and surgical complications; infections and infestations; investigations; nervous system disorders; and skin and subcutaneous tissue disorders. Specifically, CZA is commonly associated with death, pathogen resistance, thrombocytopenia, and renal failure, as well as elevated serum creatinine, diarrhea, encephalopathy, and respiratory failure. It is worth noting that Zhang et al. explicitly pointed out that pathogen resistance is the strongest AE signal in CZA therapy (Zhang et al., 2024). Data from Phase II/III clinical trials showed that the most common AEs were positive direct antiglobulin tests, nausea, and diarrhea (Shirley, 2018). Overall, as the clinical application population expands and the duration of use increases, the AE profile of CZA shows a trend toward increased severity, shifting from primarily gastrointestinal adverse reactions in the early stages to life-threatening outcomes such as nephrotoxicity, neurotoxicity, and resistance.

### 4.2 Significant AEs detected by four methods

Based on the analysis results, we found that the following AEs were significant in all four detection methods and occurred more than 20 times, including death, pathogen resistance, septic shock, decreased platelet count, renal failure, acute kidney injury, increased blood creatinine, multiple organ dysfunction syndrome, renal impairment, and encephalopathy. It broadly covers renal and urinary system diseases, blood and lymphatic system disorders, nervous system diseases, and infections and infestations.

Interpretation of treatment failure and disease progression signals. Although death and septic shock reached the threshold for signal detection, these findings likely mirror the underlying clinical status of the patients. As CZA is frequently reserved for salvage therapy in high-risk infections, these reports often represent disease severity or treatment failure rather than a direct pharmacological reaction. Acknowledging this protopathic bias is essential for a balanced safety assessment and prevents the misattribution of illness-related complications to the drug’s inherent toxicity.

The mechanism between CZA and hematological and lymphatic system disorders, particularly thrombocytopenia, primarily involves three aspects. Of paramount importance is the effect of bone marrow suppression. Ceftazidime has been demonstrated to directly inhibit the proliferation and differentiation of bone marrow megakaryocytes, thereby reducing platelet production (Nie et al., 2021). This dose-dependent effect is closely related to drug accumulation caused by renal insufficiency. Second, ceftazidime would bind to platelet membrane protein and interact with platelet antigen (Loo et al., 2012; Ali et al., 2020). These antibodies then form antibody-drug conjugates, which activate the complement system or macrophage phagocytosis, accelerating platelet destruction. Furthermore, although avibactam does not possess any intrinsic capacity for direct bone marrow suppression, it does inhibit renal tubular organic anion transporters (OAT1/3), thereby delaying ceftazidime excretion and increasing its exposure concentration in bone marrow and blood (Vishwanathan et al., 2014). This, in turn, results in an indirect enhancement of its toxicity to platelets. Hematological and lymphatic system disorders typically occur 5–14 days following administration, presenting as mild to moderate thrombocytopenia, which may resolve within one to two weeks of discontinuation. Nevertheless, for high-risk populations, such as those with renal impairment or concurrent use of other nephrotoxic medications, closer monitoring of blood counts is necessary.

The occurrence of renal dysfunction and urinary tract disorders, including acute kidney injury and elevated creatinine levels in patients treated with CZA, is primarily attributable to the direct tubular toxicity of the ceftazidime component, crystal deposition, and immune-mediated injury (Shi et al., 2024). Ceftazidime has been observed to accumulate in renal tubular epithelial cells, resulting in the inhibition of mitochondrial complex I activity, the induction of oxidative stress, and the formation of crystals in acidic urine (Shi et al., 2024). This phenomenon is attributed to the significant decrease in the solubility of ceftazidime at urine pH values below 5.5, consequently leading to tubular cell necrosis and obstruction. Furthermore, ceftazidime, as a hapten, has been demonstrated to induce acute interstitial nephritis, characterized by lymphocytic infiltration and eosinophilic urine. The risk factors for renal dysfunction and urinary tract disorders include oliguria, high-dose administration, renal insufficiency, and concomitant use of nephrotoxic drugs. Therefore, strict dose adjustment, forced hydration, and monitoring of creatinine levels are prerequisites for its clinical application.

For nervous system diseases, ceftazidime has been observed to penetrate the blood-brain barrier, thereby competitively inhibiting the binding of the central inhibitory neurotransmitter gamma-aminobutyric acid (GABA) to its receptors (Pingue et al., 2020; Vanneste et al., 2024). This results in abnormal increases in neuronal excitability, manifesting in symptoms such as delirium, myoclonus, and seizures. In patients diagnosed with meningitis, the cerebrospinal fluid/serum concentration ratio of CZA reaches 0.35–0.59, indicating its capacity to effectively penetrate the blood–brain barrier in inflammatory states (Xu et al., 2024). This characteristic confers therapeutic advantages in the treatment of central nervous system infections but also increases the risk of neurotoxicity.

### 4.3 Analysis of subgroup results

From the subgroup results, younger patients who received CZA had lower risks of hypotension, delirium, and death. This may be attributed to the younger population having fewer comorbidities, stronger drug metabolism capabilities, and better immune function, thereby reducing the risk of serious adverse events, such as delirium caused by drug accumulation-induced neurotoxicity. However, the investigation revealed no clear high-risk events in elderly patients. It was hypothesized that this was due to the absence of age data in more than 60% of the reports, resulting in the identification of no statistically significant AE. In the gender subgroup, males who received CZA had a higher risk of death, acute kidney injury, sepsis, and epilepsy. The higher risk in males may stem from the interplay of multiple factors. For instance, differences in pharmacokinetics/pharmacodynamics, such as higher average body weight and greater muscle mass in males, may lead to variations in drug distribution volume and clearance, thereby affecting effective drug exposure or increasing renal burden (Nicolson et al., 2010). The prevalence and severity of underlying conditions may be higher in male patient populations, and unhealthy lifestyle habits are more common, factors that are themselves associated with increased risks of adverse outcomes. Additionally, existing evidence suggests that gender influences immune responses, and males may have distinct regulatory mechanisms for inflammatory responses to certain infections, potentially impacting the onset and progression of sepsis (Klein and Flanagan, 2016). Therefore, it is recommended that blood counts, renal function, and neurological symptoms be closely monitored in male and elderly patients during treatment with CZA.

### 4.4 Analysis of CZA versus CZA in combination with meropenem

Descriptive analysis indicated elevated reporting rates for respiratory disorders in patients receiving CZA combined with meropenem. While signals for death and septic shock likely reflect confounding by indication (i.e., higher baseline severity in patients with metallo-ive analysis indicate CRE), the risk of respiratory failure remained distinctly elevated (aOR 3.26) after multivariable adjustment.

Crucially, the lack of a comparable signal for acute kidney injury (aOR 1.07) argues against the hypothesis that respiratory failure is merely a secondary consequence of renal toxicity (Steffens et al., 2021). Instead, this dissociation suggests a specific pharmacodynamic interaction, likely involving synergistic neuromuscular blockade. Meropenem is known to antagonize GABA receptors, increasing neuronal excitability, while ceftazidime can inhibit presynaptic acetylcholine release by chelating calcium ions at the neuromuscular junction (Zhanel et al., 2009). Concomitant use may potentiate this blockade, leading to respiratory muscle weakness, particularly in patients with drug accumulation.

Additionally, immune-mediated mechanisms cannot be excluded. As ceftazidime acts as a hapten and meropenem can induce histamine release, the combination could theoretically amplify the risk of acute immune-mediated pneumonitis and alveolar edema. Collectively, these findings highlight a specific safety signal that warrants vigilant monitoring of respiratory status during combination therapy.

## 5 Limitations

Our study has several limitations, primarily stemming from the inherent nature of the FAERS database as a spontaneous reporting system. First, the data are influenced by reporting biases, including under-reporting, duplicate records, and systematic distortions like the Weber effect and notoriety bias, potentially compromising information completeness and accuracy. Second, reports frequently lack granular clinical details has several laboratory values, imaging findings, and comprehensive medical histories verily limiting the depth of clinical assessment. Third, the analysis is susceptible to “confounding by indication,” where adverse events arising from the underlying pathology (e.g., complications of severe infection) may be indistinguishable from drug-induced toxicity. Additionally, while we performed multivariable logistic regression to adjust for age, sex, and concomitant nephrotoxins, other potential confounders — such as specific comorbidities and granular disease severity scores (e.g., APACHE II)—could not be fully captured due to database limitations. Finally, since the total number of patients exposed to CZA remains unknown (denominator lacking), absolute incidence rates of adverse events cannot be determined. Consequently, the identified signals represent statistical associations rather than established biological causality, particularly given the absence of rigorous adjudication for individual reports using standard tools like the Naranjo Scale. Therefore, prospective cohort studies or clinical trials are essential for further validation.

## 6 Conclusion

This study employed pharmacovigilance analysis to assess the potential AEs of CZA using the FAERS database. The most commonly reported AEs include death, pathogen resistance, septic shock, decreased platelet count, renal failure, pyrexia, and acute kidney injury, emphasizing the need for monitoring and management during its use. Particularly, some newly identified AEs, including multiple organ dysfunction syndrome, respiratory failure, and hypotension, should be carefully consideration. The post-marketing safety information for CZA contributes to minimizing the risks associated with its use.

## Data Availability

The datasets presented in this study can be found in online repositories. The names of the repository/repositories and accession number(s) can be found below: https://fis.fda.gov/extensions/FPD-QDE-FAERS/FPD-QDE-FAERS.html.

## Supplementary material

Supplementary Table 1 The READUS-PV checklist.

Supplementary Table 2 Multivariable logistic regression analysis of clinical outcomes associated with CZA combination therapy compared to monotherapy.
