# Supplementary material for: Real-world study of adverse events associated with ceftazidime/avibactam based on the U.S. Food and Drug Administration adverse event reporting system database
Source: Front Cell Infect Microbiol. 2026 Jan 22;16:1698293. doi: 10.3389/fcimb.2026.1698293 (PMC12872935; doi:10.3389/fcimb.2026.1698293)
Supplement: Supplementary Table 1 — The READUS-PV checklist. [file Table1.docx]

| Supplementary Table S1. The READUS-PV Checklist | | | |
| --- | --- | --- | --- |
| Section and topic | Item # | Checklist item | Location where item is reported |
| Title |  |  |  |
|  | 1a | If disproportionality analyses are a prominent component of the published study, the study should be identified as a “disproportionality analysis”. The type of data and name of the database(s) should be specified. | *Title Page (Title & Abstract)* |
|  | 1b | Report the name of adverse event(s) and/or drug(s) under study, when applicable. | *Title Page* |
| Introduction |  |  |  |
| Background | 2a | Describe the drug(s) and its utilization, the nature of the adverse event(s) under study and its frequency, and the existing knowledge on the drug-event combination. | *Introduction* |
|  | 2b | Specify the rationale for performing the analysis, e.g., as part of routine pharmacovigilance, to investigate an overall safety profile, or to assess a pre-specified hypothesis. | *Introduction* |
|  | 2c | Explain why ICSR databases and disproportionality analysis are suitable to fill the knowledge gap. | *Introduction* |
| Objectives | *3* | State specific objectives, identifying the adverse event(s), the drug(s), and the reference group, including any pre-specified hypothesis, if applicable. | *Introduction (Last paragraph)* |
| Methods |  |  |  |
| Study design | 4a | Identify the study (i.e., “disproportionality analysis”) and the type of data used (e.g., “individual case safety reports”). | *Section 2.1 & Section 2.3* |
|  | 4b | Provide an outline of the entire study design, including primary and sensitivity analyses performed, and other designs such as case-by-case analysis or literature review. | *Section 2; Figure 1* |
| Data description, access, and pre-processing | 5a | Specify the name of the database(s), the database(s) custodian, and the coverage. Specify the type/number of drugs included within the database and the thesaurus, taxonomies, or ontologies used for coding drugs and events. | *Section 2.1* |
|  | 5b | Specify the extraction dates and describe and justify all choices used for data pre-processing, including any data transformation or exclusion, if appropriate. | *Section 2.1 & Section 2.2* |
| Variables definition | 6a | Describe the study population, including any restriction. | *Section 2.1; Table 1 (Demographics)* |
|  | 6b | Describe the nature and the meaning of key variables assessed in the work. | *Section 2.1* |
|  | 6c | Specify and justify any grouping of drugs or events. For drugs, specify and justify whether active ingredients/trade names/salts were considered and/or the selected role. | *Section 2.1 (Search terms & MedDRA)* |
|  | 6d | Describe any additional data source used, the type of data, and how they interact with ICSRs. | *N/A (Only FAERS was used)* |
| Statistical methods | 7a | Present any descriptive analysis performed, specifying variables investigated, statistical tests, and significance thresholds. | *Section 2.3; Table 1* |
|  | 7b | Describe the measure(s) selected for the disproportionality analysis including any threshold used to identify signals of disproportionate reporting. Explain the reason for this choice if applicable. | *Section 2.3 (ROR, PRR, BCPNN formulas)* |
|  | 7c | Clearly describe any sensitivity analysis and any tool to control confounding, including any restriction, subgroup, stratification, adjustment, or interaction. | *Section 2.3 (Subgroup analysis methods)* |
|  | 7d | Specify the variables and methods used for the case-by-case analysis, including any algorithm or criteria used to assess causality, if performed. | *N/A (Not performed)* |
|  | 7e | Specify any statistical methods used for other data sources. | *N/A* |
| Results |  |  |  |
| Participants | 8a | Specify the number of individual case safety reports included at each stage, including reasons for exclusion. | *Section 3.1; Figure 1* |
|  | 8b | Provide key demographic and clinical characteristics of cases, if possible comparing cases with any appropriate reference group. | *Section 3.1; Table 1* |
| Disproportionality analysis | *9* | Present all results including confidence intervals. Present also results of sensitivity analyses, if performed. | *Section 3.2; Table 2–5; Figure 2–7* |
| Case-by-case analysis | *10* | Present the case-by-case analysis of key variables. Present the causality assessment, if applicable. | *N/A* |
| Discussion |  |  |  |
| Key results | *11* | Discuss key results with reference to study objectives and contextualize them within the current literature and other consulted sources. Clearly discriminate between expected reactions and emerging safety signals. | *Discussion (Sections 4.1 – 4.4)* |
| External validity | 12a | Discuss the external validity of the results to the general population. | *Discussion* |
|  | 12b | Discuss the potential relevance of results in clinical practice | *Discussion; Conclusion* |
|  | 12c | Propose further study designs if applicable | *Discussion (Limitations section)* |
| Limitations | *13* | Present general limitations, making clear that disproportionality analysis alone cannot prove causation or measure incidence, and specific limitations, including confounding and reporting bias and efforts to mitigate them. | *Discussion (Section 5, Limitations)* |
| Declarations |  |  |  |
|  | 14a | Provide the source of funding/sponsorship and the role of the funders/sponsors for the present study and for any original study on which the present article is based. | *Funding Section* |
|  | 14b | Clearly identify potential commercial and intellectual conflicts of interest (e.g., link to any drug/event investigated, whether financial, legal action, or software used). | *Declaration of interest Section* |
|  | 14c | Declare any institutional approval needed or granted in the investigation. | *Section 2.1 (Ethics statement)* |
|  | 14d | Include a statement on data availability, code availability (including the version of the statistical software used), and protocol registration. | *Data availability statement Section* |
