# Supplementary material for: Real-world study of adverse events associated with ceftazidime/avibactam based on the U.S. Food and Drug Administration adverse event reporting system database
Source: Front Cell Infect Microbiol. 2026 Jan 22;16:1698293. doi: 10.3389/fcimb.2026.1698293 (PMC12872935; doi:10.3389/fcimb.2026.1698293)
Supplement: Supplementary Table 2 — Multivariable logistic regression analysis of clinical outcomes associated with CZA combination therapy compared to monotherapy. [file Table2.docx]

Supplementary Table S2. Multivariable logistic regression analysis of clinical outcomes associated with CZA combination therapy compared to monotherapy.

| Outcome | Group | Total N | Events, n (%) | aOR (95% CI) | P-value |
| --- | --- | --- | --- | --- | --- |
| Respiratory Failure | Monotherapy | 986 | 13 (1.3%) | 1.00 (Ref) | - |
|  | Combination | 96 | 6 (6.3%) | 3.26 (1.07 – 9.93) | 0.038* |
| Acute Kidney Injury | Monotherapy | 986 | 78 (7.9%) | 1.00 (Ref) | - |
|  | Combination | 96 | 10 (10.4%) | 1.07 (0.50 – 2.29) | 0.868 |
| Septic Shock | Monotherapy | 986 | 23 (2.3%) | 1.00 (Ref) | - |
|  | Combination | 96 | 9 (9.4%) | 2.93 (1.16 – 7.38) | 0.023* |
| Death | Monotherapy | 986 | 305 (30.9%) | 1.00 (Ref) | - |
|  | Combination | 96 | 40 (41.7%) | 2.07 (1.30 – 3.31) | 0.002* |

Abbreviations: aOR, adjusted odds ratio; CI, confidence interval; CZA, ceftazidime/avibactam.

The multivariable logistic regression model was adjusted for age, sex, reporting year, and concomitant use of nephrotoxic antibiotics (specifically vancomycin and aminoglycosides).

* P < 0.05 indicates statistical significance.
